# Towards an advanced testing strategy for genotoxicity using image-based 2D and 3D HepG2 DNA damage response fluorescent protein reporters

**DOI:** 10.1093/mutage/geab031

**Published:** 2021-08-27

**Authors:** Bas ter Braak, Marije Niemeijer, Liesanne Wolters, Sylvia Le Dévédec, Peter Bouwman, Bob van de Water

**Affiliations:** Division of Drug Discovery and Safety, Leiden Academic Centre for Drug Research, Leiden University, Leiden, The Netherlands

## Abstract

*In vitro* assessment of mutagenicity is an essential component in the chemical risk assessment. Given the diverse modes of action by which chemicals can induce DNA damage, it is essential that these *in vitro* assays are carefully evaluated for their possibilities and limitations. In this study, we used a fluorescent protein HepG2 reporter test system in combination with high content imaging. To measure induction of the DNA damage response (DDR), we used three different green fluorescent protein reporters for p53 pathway activation. These allowed for accurate quantification of p53, p21 and BTG2 (BTG anti-proliferation factor 2) protein expression and cell viability parameters at a single cell or spheroid resolution. The reporter lines were cultured as 2D monolayers and as 3D spheroids. Furthermore, liver maturity and cytochrome P450 enzyme expression were increased by culturing in an amino acid-rich (AAGLY) medium. We found that culture conditions that support a sustained proliferative state (2D culturing with normal DMEM medium) give superior sensitivity when genotoxic compounds are tested that do not require metabolisation and of which the mutagenic mode of action is dependent on replication. For compounds, which are metabolically converted to mutagenic metabolites, more differentiated HepG2 DDR reporters (e.g. 3D cultures) showed a higher sensitivity. This study stratifies how different culture methods of HepG2 DDR reporter cells can influence the sensitivity towards diverse genotoxicants and how this provides opportunities for a tiered genotoxicity testing strategy.

## Introduction

In the current chemical risk assessment, the evaluation of genotoxicity is quite different from other chemical-induced adverse reactions as the typical ‘safe threshold analysis’ is thought to be not applicable to genotoxicants. This is because even low doses of genotoxicants can lead to DNA damage and thereby increase the chance to develop malignancies. However, besides directly damaging DNA, genotoxic compounds may also interfere with specific cellular processes (e.g. DNA synthesis or repair), thereby having indirect genotoxic effects. For compounds with such indirect genotoxic mode of action, it is relevant to determine the concentration level where no DNA damage effects occur (1).

Several well-established test systems are currently being used to identify potential genotoxic effects of chemicals. With the prokaryotic Ames test, specific mutagenic events, such as frameshifts or point mutations, can be observed using specific *Salmonella* tester strains. Although this test system is fast, inexpensive and easy to use, it suffers from poor sensitivity, likely due to differences in the response to drugs and DNA damage in prokaryotes versus higher eukaryotes (2). Another well-known genotoxicity test system is the Comet assay developed in 1988 by Singh and colleagues (3), which can detect DNA strand breaks in individual eukaryotic cells by quantifying the DNA track on an agarose gel. Since then many advances have been made to the standard Comet assay, so that it can identify different types of DNA damage (4,5). However, this highly sensitive assay is still difficult to standardise because of a lack of consensus and potentially suffers from a relative high rate of false positives due to cytotoxicity effects (6,7). Besides, the comet assay can only be used to determine if exposure to a compound results in DNA breaks, other types of DNA damage (e.g. point mutations) are not revealed (2). A third genotoxicity test, which is also recommended by the OECD guidelines for chemical testing, is the Micronucleus test. Micronuclei are cytoplasmic bodies containing either a portion of an acentric chromosome or lagging whole chromosomes, a result of incorrect chromosome segregation during anaphase. The micronuclei can be quantified and are a measure for genotoxicity (8). Limitations of this assay are that a cell division is required in the test cells, the relatively low specificity and the fact that gene mutations cannot be detected with this assay (2). In the last few decades, several variants of the Ames test, Comet assay and Micronucleus test have been developed to improve throughput, human relevance and robustness, but concerns on limited predictivity remain.

A new type of genotoxicity testing does not focus on quantification of chemically induced genomic injury itself, but on the quantification of the cellular mechanisms that repair this DNA damage. It is expected that this strategy results in test methods with a higher sensitivity, as these repair mechanisms are already induced at low levels of DNA damage. Furthermore, for the different types of genotoxicity, different repair mechanisms can be identified, so that the chemical mode of action by which the damage is induced can be eluded (1). The simplest variants of these assays comprise of antibody fluorophore stainings, targeting focal accumulation of biomarker proteins involved in DNA damage repair of which γH2AX, 53BP1 and RAD51 are examples (9). Lack of robustness and the limitation of a single end point measurement in fixed cells are major drawbacks of antibody-based methods. A more sophisticated method would be to use fluorescent reporter models (10). The GADD45a-based fluorophore and luciferase reporter assays, GreenScreen and BlueScreen, are applied for high-throughput genotoxic hazard identification (11). With a FACS-based measurement the ToxTracker can quantify chemically induced DNA damage repair using reporter stem cell lines. By using a combination of different reporter lines, this technology is able to detect both direct and indirect genotoxic effects and it can discriminate between clastogenic and aneugenic modes of action (12,13).

We have previously developed a large panel of fluorescent reporters for adaptive cellular stress responses, including the DNA damage response (DDR) in the human liver cancer cell line, HepG2 (10,11). We have applied these reporters for the prediction of drug-induced liver injury (14–16). In these reporter cell lines, chemical induction activates expression of fluorescent-tagged stress biomarker proteins. By combining live cell confocal imaging with an automated image segmentation pipeline, the biomarker expression level as well as its subcellular localisation can be accurately quantified over time and at a single cell level. Besides 2D culturing, these cell lines can also be cultured as spheroids in a 3D matrigel environment (17,18). These spheroids are stable for at least 2 weeks, which allows for repeated dosing. Furthermore, the HepG2 spheroids are metabolically more active as compared to HepG2 cells grown on a 2D surface, showing physiologically relevant levels of cytochrome P450 enzymes (17). Most chemicals are metabolised in the human liver, which either leads to toxic clearing or the production of toxic metabolites; therefore, the presence of drug-metabolising enzymes in a test system is an important feature (19). Of interest, when HepG2 cells are cultured in an amino acid-rich (AAGLY; Amino Acid-rich normal DMEM medium supplemented with 2% GLYcine) medium they change their glucose-dependent metabolism to an amino acid-fuelled profile, thus promoting further differentiation and expression of P450 enzymes at levels similar to primary human hepatocytes (PHHs), even in a 2D environment (20).

Here, we systematically investigated the application of p53 pathway DDR reporters in human liver models with different levels of maturation and metabolic competence. We used three HepG2 DNA damage green fluorescent protein (GFP) reporter cell lines; p53-GFP, p21-GFP and BTG2-GFP. These reporter cell lines were cultured in four different conditions: 2D using conventional Dulbecco’s Modified Eagles’s Medium (DMEM) or AAGLY medium, and 3D using DMEM/F12 or AAGLY medium. To assess sensitivity of the different culture conditions towards DNA damage, we exposed these models to five different chemicals that are known to cause a direct or indirect genotoxic effect with a distinct mode of action (Table 1). We envision that this study contributes to a better understanding of the different culture conditions that impact on metabolic competence and assay sensitivity in the context of the DDR. This will allow refinement of chemical safety testing strategies for a better-informed risk evaluation.

**Table 1.** List of compounds with the concentration range used in this study

Compound	Abbreviation	Tested concentration range
Aflatoxin B1	AFB	0.10–21.54 µM
*1. Aflatoxin B1 is converted into reactive intermediate AFB1-8,9-epoxide mainly by CYP3A4.* *2. AFB1-8,9-epoxide forms DNA adducts.* *3. DNA damage is induced with direct compound–DNA interaction* (21).		
Brequinar	BRE	0.22–100 µM
*1. Brequinar inhibits dihydroorotate dehydrogenase (DHOD).* *2. Pyrimidine biosynthesis is blocked.* *3. DNA/RNA synthesis and repair is inhibited.* *4. DNA damage is induced indirectly without compound–DNA interaction* (22).		
Cisplatin	CIS	0.10–21.54 µM
*1. Cisplatin is taken up by the cell by copper transporters.* *2. Cisplatin cross-links (mainly intrastrand) with the purine bases on the DNA.* *3. Interference with DNA repair mechanisms.* *4. DNA damage is induced with direct compound–DNA interaction* (23,24).		
Gemcitabine	GEM	0.0037–0.80 µM
*1. Gemcitabine is phosphorylated by deoxycytidine kinase to its active form.* *2. Gemcitabine diphosphate or triphosphate (dFdCTP) incorporates at the end of the elongating DNA strand.* *3. RAD51-dependent homologous recombination and DNA synthesis is inhibited.* *4. DNA damage is induced with direct compound–DNA interaction* (25).		
Mitomycin C	MMC	0.10–21.54 µM
*1. Mitomycin C makes DNA cross-links (both inter- and intrastrand).* *2. DNA damage is induced with direct compound–DNA interaction.* *1. Cytochrome P450 reductase induces redox cycling of mitomycin C.* *2. ROS are formed.* *3. ROS leads to an indirect DNA damage effect* (24,26).		

The genotoxic mode of action based on literature.

## Materials and Methods

### Chemicals or reagents

Mitomycin C and aflatoxin B1 were obtained from Sigma-Aldrich (Zwijndrecht, The Netherlands). Gemcitabine was acquired from Eli Lilly (Indianapolis, IN, USA) and brequinar was obtained from Fluorochem (Hadfield, UK). Cisplatin was acquired from Ebewe Pharma (Unterach am Attersee, Austria). Compounds were dissolved in dimethylsulfoxide (DMSO), except for mitomycin C [phosphate-buffered saline (PBS)], cisplatin (PBS) and gemcitabine (sterile H_2_O). The maximum final DMSO concentration used was 0.2% (v/v).

### Cell culturing

Human hepatocellular carcinoma HepG2 cells were obtained from the American Type Tissue Culture Collection (ATCC, Wessel, Germany). The HepG2 BAC-GFP reporters for human *BTG2*, *p21* and *p53* were generated previously and used to monitor the DDR (14,18). Cells were maintained in DMEM high glucose (Gibco) supplemented with 10% (v/v) fetal bovine serum (FBS), 25 U/ml penicillin and 25 µg/ml streptomycin (referred to as normal DMEM medium) at 37°C and 5% CO_2_. The reporter cells were tested between passages 19–21 throughout the data presented (Supplementary Figure 1, available at *Mutagenesis* Online).

### 2D cell culturing in normal DMEM medium

Cells were plated in Greiner Bio-One black SCREENSTAR 384-well plates (Alphen aan den Rijn, The Netherlands), at 10 000 cells per well in normal DMEM medium. Cells were allowed to adhere for 24 h before treatment or RNA isolation.

### 2D cell culturing in AAGLY medium

Cells were plated in Greiner Bio-One black µClear 384-well plates (Alphen aan den Rijn, The Netherlands), at 18 000 cells per well in normal DMEM medium. Forty-eight hours after plating, cells switched from normal DMEM medium to AAGLY medium consisting of DMEM low glucose supplemented with 7.7% (v/v) FBS, 20 U/ml penicillin, 20 µg/ml streptomycin, 160 µl/ml MEM non-essential amino acids solution (100×), 80 µl/ml MEM essential amino acids (50×) solution and 2% glycine with a pH of 7 (20). To drive hepatic differentiation, cells were cultured for 30 days before treatment or RNA isolation. Medium was refreshed twice a week.

### 3D cell culturing in DMEM/F12 medium

3D culturing was performed as described previously (17,18). Briefly, cells were cultured in a layer of 5 mg/ml Matrigel matrix basement membrane, growth factor reduced (Corning, Cat#354230, Lot#6130005) in Greiner Bio-One black µclear 384-well plates (Alphen aan den Rijn, The Netherlands) at a density of 1000 cells per well to form liver spheroids in 21 days before treatment or RNA isolation. Spheroids were maintained in DMEM/F12 high glucose and phenol red free supplemented with 10% FBS and 25 U/ml penicillin and 25 µg/ml streptomycin (referred to as DMEM/F12 medium). Medium was refreshed twice a week.

### 3D cell culturing in AAGLY medium

3D culturing was performed as described above. However, 7 days post seeding, DMEM/F12 medium was replaced by AAGLY medium with no phenol red. AAGLY medium was then refreshed twice a week until 21 days before treatment or RNA isolation.

### Cell treatment and viability

**Fig. 1.**
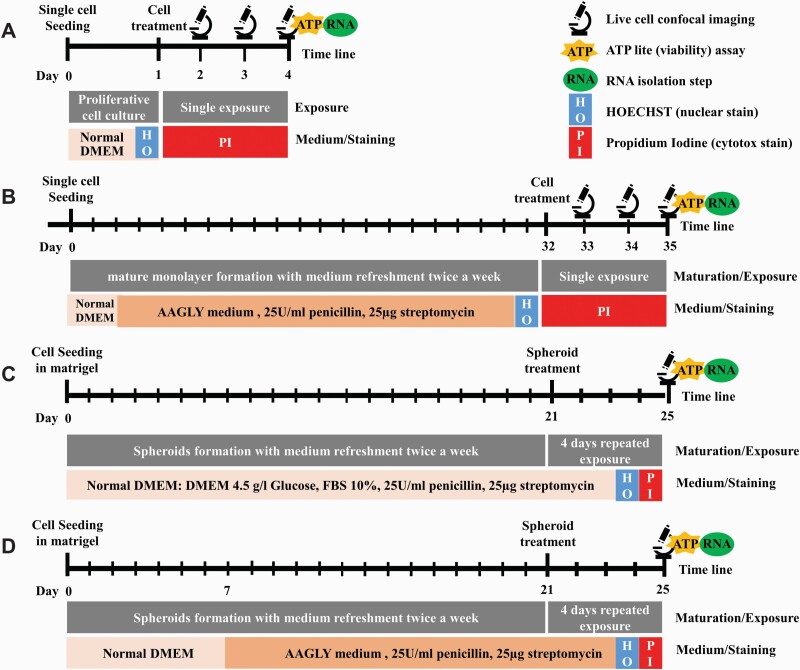
Overview of the four different HepG2 models used in this study. Schematic overview of the experimental procedures. The name of the different models are indicated in the upper right corner: HepG2 cultured in 2D and normal DMEM medium (A), HepG2 cultured in 2D and AAGLY medium (B), HepG2 cultured in 3D and normal DMEM medium (C), HepG2 cultured in 3D and AAGLY medium (D).

Two different compound exposure scenarios were performed: a single exposure in 2D and a 4-day repeated exposure in 3D. A schematic overview of the tested exposure scenarios for each HepG2 model has been depicted in Figure 1. For the single exposure scenario in 2D, medium was replaced by freshly diluted compound in medium 24 h post seeding. DDRs were monitored after 24, 48 and 72 h by live cell confocal imaging (Figure 1A and B). For the 4-day repeated exposure scenario in 3D, each day medium was replaced by freshly diluted compound in medium for four consecutive days (Figure 1C and D). The imaging was started 24 h after the last exposure. For both scenarios, five compounds in eight concentrations were tested. DMSO, PBS or ultrapure H_2_O were used as solvent controls. The ATP-lite luminescence kit (Perkin Elmer) was used according to supplier’s protocol to measure cell viability. Measurements were performed 72 h or 24 h post single or repeated exposure, respectively. Absolute IC_50_ values have been calculated over the ATP-lite data by determining the intersect of the fitted concentration–response curve with the 50% viability baseline via GraphPad prism 8.1.1.

### Cell imaging

Prior to imaging, cells were stained with Hoechst 33342 at a concentration of 0.1 µg/ml to visualise the nuclei. To examine compound-induced cell death using confocal microscopy, propidium iodide (PI) was added during all compound exposures at a concentration of 100 µM to stain for necrotic or late apoptotic cells. The induction of GFP intensities and PI were monitored with a Nikon Eclipse Ti confocal laser microscope (Nikon, Amsterdam, The Netherlands), equipped with lasers at wavelengths 408, 488 and 561, an automated stage and perfect focus system at 37°C and 5% CO_2_. Images for 2D cultures were acquired with a Nikon 20x Dry Plan Apo VC NA 0.75 objective. Images for 3D cultures were acquired with a Nikon 10x Dry Plan Fluor NA 0.3 objective. For each condition, a z-stack of 9–11 images was generated with a step size of 30 µm.

### Quantitative image analysis

For 2D cultures, image quantification was performed with Cell Profiler version 2.2.0 (Broad Institute, Cambridge, USA) using modules described previously (14). Nuclei segmentation based on Hoechst was done in ImageJ using an in-house developed macro based on the watershed masked clustering algorithm (27). Segmentation of the cytoplasmic area was done using the Distance N method expanding the edges of nuclear objects with six pixels followed by subtraction of the nuclear area. p21 and p53-GFP intensities were measured in the nuclei and BTG2-GFP in the cytoplasm. The PI-positive cells were identified by an overlay of the PI signal and the segmented nuclei. A cell was considered positive when there was at least 10% overlap. Results were stored in HDF5 format (28). To extract summarised data for further analysis and visualisation, in-house developed R-scripts were used in RStudio (version 1.0.153) (Boston, MA, USA) (29–31). The mean nuclear and cytoplasmic intensities of all measured single cells and the fraction of PI-positive cells for each image were extracted as output from the HDF5 files.

For 3D cultures, the NIS Elements analysis software (Nikon, Amsterdam, The Netherlands) was used to quantify the GFP intensity and PI-positive area within spheroids. First, a 2D projection per replicate treatment was created based on the maximum Hoechst intensity across z-stacks. Then, spheroids were segmented by setting a threshold for the Hoechst signal. The GFP intensity was measured in the spheroids and the PI-positive area within spheroids was determined by the overlap of PI signal within spheroids. Further analysis was performed using in-house developed R-scripts in RStudio (version 1.0.153) (Boston, MA, USA).

To compare the GFP induction of the tested models, first GFP intensities were min-max normalised for each model. Then, a hierarchical clustering analysis was performed using R package pheatmap (32). First, Euclidean distances were calculated between different models, reporters and exposure durations. Thereafter, the mean of the Euclidean distances for the three different reporters for each model and exposure duration was used for hierarchical clustering using the complete linkage method. For the end points for cell death, a hierarchical clustering was performed based on Euclidean distance between each model, exposure duration and end point.

### Real-time qPCR

Total RNA from the 2D and 3D cultures was isolated each from eight wells of a 384-well plates using the NucleoSpin® mRNA isolation kit or Trizol reagent (Invitrogen) according to manufacturer’s instructions, respectively. Isolated RNA was considered of sufficient quality when A260/280 and A260/230 ratios were higher than 1.9 and 1.5, respectively. The RevertAid first-strand cDNA synthesis kit (Thermo Fisher) with Oligo(dT) 18 primers was used to generate the template from 500 ng RNA for the real time quantitative PCR (RT-qPCR) experiments. SYBR green master mix (Thermo Fisher) was used as a dye to monitor the accumulation of the PCR product using 25 ng cDNA template. In Supplementary Table 1 (available at *Mutagenesis* Online), the primers sequences can be found that were used in the PCR reaction; denaturation (95°C for 30 s), annealing (60°C for 1 min), extension (72°C for 30 s) with 40 cycles. The 2^−ΔΔCT^ method was used to quantify relative gene expression profiles using *GAPDH* identified as a stable housekeeping gene for HepG2 (33) and the HepG2 2D model as a reference sample (34).

### TempO-seq transcriptomics

Similarity between HepG2 wild-type and DDR reporter cells in p53 signalling was evaluated by measuring the mRNA expression of a targeted gene set consisting of the S1500+ gene list (35) using the TempO-seq technology by BioSpyder Technologies Inc. (Carlsbad, CA, USA) (36). In brief, HepG2 cells were seeded in 96 wells plates (Corning, Amsterdam, The Netherlands) at a density of 156 000 cells/cm^2^. Next day, cells were exposed to cisplatin in a wide concentration range for 8 or 24 h followed by sample collection using 1× BNN lysis buffer (BioSpyder). Samples were lysed for 15 min at room temperature, stored at −80°C and shipped for transcriptome analysis by BioSpyder. The TempO-seqR package was used for the alignment of raw reads, followed by normalisation using DESeq2 R package (37) of the read counts and log2 transformed.

### Data analysis

Point of departures (PODs) have been determined using an in-house established R package ‘modelpod’. Concentration–response curves are fitted with Loess regression using the loess function from the base stats R package (with a span of 2/4 and 1 polynomial degree). The intersect of this fitted curve with the sum of the DMSO control value and two times the standard deviation (SD) from the regression of the DMSO gives an X value, the lowest concentration at which we observe a significant (positive or negative) effect defined as the PoD.

### Statistics

For all experiments three independent biological replicates were performed. Additionally, for the imaging of the 2D models two positions per well have been imaged which were treated as technical replicates. Error bars in the concentration–response plots represent the SD of the three biological replicates. Significance for quantitative PCR (qPCR) data was determined using two-way ANOVA with Tukey’s multiple testing correction represented as *P_adj_ < 0.05, **P_adj_ < 0.01 and ***P_adj_ < 0.001.

## Results

### Characterisation of hepatic phenotype of HepG2 models

Previously, we generated the HepG2 BAC-GFP reporter platform as a tool for the assessment of chemical-induced toxicity. Using live cell confocal microscopy, we accurately quantified stress response activation of a total of eight different stress response pathways using 20 different biomarkers (14–16). In this study, we focussed on BTG2, p21 and p53 reporters of the DDR. Since differences in drug metabolism capacity can have profound genotoxic consequences, we tested different 2D and 3D culture set-ups to study their suitability for the recognition of chemical-induced DNA damage.

**Fig. 2.**
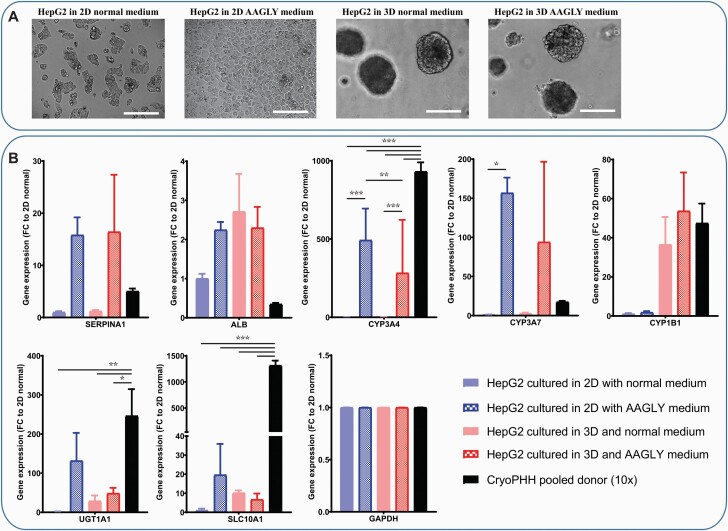
Hepatocyte maturation status of four different HepG2 models. (A) Bright field pictures showing morphological differences between the different models; HepG2 cultured in 2D and normal DMEM medium, HepG2 cultured in 2D and AAGLY medium, HepG2 cultured in 3D and normal DMEM medium, HepG2 cultured in 3D and AAGLY medium from left to right. (B) Gene expression profiles of some hepatocyte markers (*SERPINA1* and *ALB*), CYP enzymes (*3A4*, *3A7* and *1B1*) and transporters (*UGT1A1* and *SLC10A1*) and the housekeeping genes (*GAPDH*). Gene expression values are relative to model 1 (HepG2 cultured in 2D and normal DMEM medium) and benchmarked to a pool of 10 different donors of cryopreserved PHHs (10×). *N* = 6; error bars represent SD; significance levels represented as *P_adj_ < 0.05, **P_adj_ < 0.01 and ***P_adj_ < 0.001.

These HepG2 reporter cells were cultured in 2D either using normal medium having limited metabolic activity or medium with high levels of amino acids (AAGLY medium) to induce hepatocyte maturation for 30 days (Figure 2A), as previously described by Boon and colleagues (20). Culturing HepG2 cells with AAGLY medium stops the proliferating capacity and allows the formation of a monolayer which was highly similar to the morphology of a PHH 2D culture (Figure 2A) (32).

Previously, we have shown that 3D culturing of the HepG2 reporter model also resulted in a liver model with an increased metabolic potential (18). By culturing HepG2 cells in a layer of Matrigel, cells clustered, stopped proliferating and formed well-rounded spheroids in 3 weeks resulting in an improved hepatic phenotype (Figure 2). We also combined the 3D culturing of the HepG2 reporter cells with the AAGLY medium. Since the AAGLY medium stopped the cell proliferation, which was necessary for spheroid formation, we used normal DMEM medium for the first 7 days. We anticipated that 2 weeks of AAGLY medium would further enhance the HepG2 hepatocyte maturation in the 3D set-up. The morphology of both 3D models was similar in the 3D AAGLY medium condition (Figure 2).

To evaluate the relative differentiation level of the different cultures, we determined several key hepatocyte differentiation markers. Culturing HepG2 cells in AAGLY medium had the greatest effect on hepatocyte maturation, with strong upregulation of hepatocyte markers *SERPINA1*, *CYP3A4*, *CYP3A7*, *UGT1A1* and *SLC10A1* as compared to normal DMEM medium (Figure 2B). However, the addition of AAGLY medium did not lead to induction of *CYP1B1* in 2D, while it was upregulated by ~50-fold in the 3D culturing conditions. In general, culturing in a 3D set-up in DMEM led to increased expression of *ALB*, *CYP1B1*, *UGT1A1* and *SLC10A1* compared to 2D in DMEM, although this upregulation was not significant due to higher variance. Combining AAGLY medium with the 3D set-up led to a clear induction of *CYP3A4*, *SERPINA1* and *CYP3A7* compared to DMEM, reaching similar levels as 2D in AAGLY medium. While the more mature models showed hepatocyte gene expression profiles similar to plated cryopreserved PHH, some genes like *SLC10A1* were still markedly lower as compared to this gold standard.

### DDR activation and quantification

Next, we tested a set of compounds (see Table 1) on the three different DDR reporters under the various culture conditions. To verify the similarity in p53 signalling between these three different DDR reporters, the expression of key related genes has been evaluated upon exposure to cisplatin for 8 or 24 h. Here, no significant difference in stress response was measured indicative of preservation of wild-type p53 signalling during reporter development and passaging (Supplementary Figure 1, available at *Mutagenesis* Online).

**Fig. 3.**
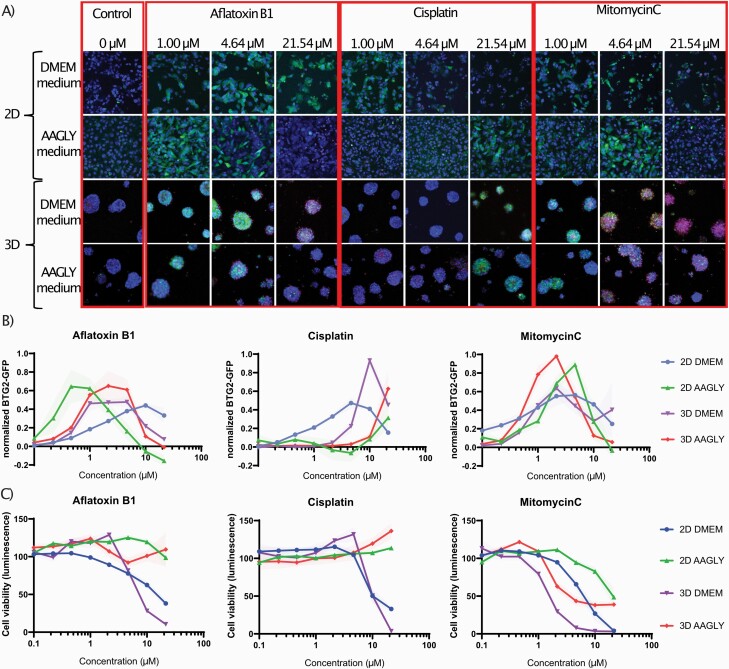
Example images of fluorophore reporter activation and corresponding concentration–response plots. (A) Representative examples of confocal images of the BTG2-GFP reporter upon activation by aflatoxin B1, cisplatin or mitomycin C in the four different culture conditions. For the 2D, only images are shown of the 72-h time point. The blue pseudocolour represents Hoechst (nuclei) staining, green represents the GFP reporter activation and red shows the PI (cell death) staining. (B) Concentration–response plots of the min-max normalised BTG2-GFP reporter activation upon treatment with aflatoxin B1, cisplatin or mitomycin C in the four different culture conditions. Fluorophore data are shown as fold changes as compared to basal (solvent) conditions. *N* = 3; the light-coloured shaded area represents error bars (SD). (C) Concentration–response plots of cell viability (ATP-lite) upon activation by aflatoxin B1, cisplatin or mitomycin C in the four different culture conditions. Fluorophore data are shown as fold changes as compared to basal (solvent) conditions. *N* = 3; the light-coloured shaded area represents error bars (SD).

Since proliferation of HepG2 cells cultured in the conventional normal DMEM medium hampered long-term culturing without passaging, only a single compound treatment was tested for 24, 48 or 72 h. Therefore, for comparison, also the AAGLY medium in 2D was evaluated using the same treatment regime. In 3D both conventional normal DMEM medium and AAGLY medium spheroids are stable and allowed for repeated 4-day exposure. DDR reporter activation was observed in all the different culture conditions and upon treatment of three different example compounds, aflatoxin B1, cisplatin and mitomycin C (Figure 3A). When exposed to the different genotoxic compounds, the BTG2-GFP was strongly induced while in control conditions no induction of BTG2-GFP was seen (Figure 3B). Importantly, the various culture conditions had a very different effect on the sensitivity towards the genotoxicants. For example, when HepG2 cells were cultured in 2D with conventional normal DMEM medium, aflatoxin B1 led to a steady concentration-dependent induction of BTG2-GFP with a peak at 10 µM. In contrast, when cultured in AAGLY medium, the HepG2 became more sensitive and the maximum BTG2 upregulation was seen at ~40 times lower concentrations. Unexpectedly, this increased sensitivity of HepG2 cells cultured in 2D with AAGLY medium with respect to BTG2-GFP activation for aflatoxin B1 did not translate to an increased sensitivity towards cell death, possibly more mature HepG2s are better equipped to counteract the aflatoxin B1-induced adversity. For direct mutagens like cisplatin, the 2D and 3D model in normal DMEM medium showed the highest BTG2-GFP sensitivity. These models were also most sensitive with the cell viability readout with this compound. For mitomycin C, we could clearly observe that at low concentrations all models showed a strong BTG2-GFP response and at high concentrations cell death was induced, visualised by the PI-positive cells/spheroids and a concentration-dependent reduction of ATP content. In contrast, the (in)direct anti-metabolites gemcitabine and brequinar only induced BTG2-GFP responses in the proliferating HepG2 cultured in 2D in normal DMEM medium (Supplementary Figure 2, available at *Mutagenesis* Online).

### Cell death induction upon chemical-induced DNA damage in HepG2 models

**Fig. 4.**
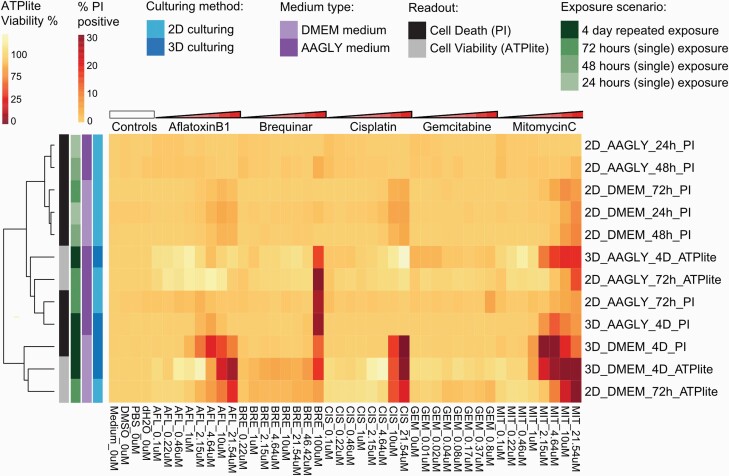
Hierarchical clustering of cell death data. Hierarchical clustering (Euclidean distance with Ward’s distance) of PI-positive fraction and ATP content (viability) upon exposure to five compounds at eight concentrations in HepG2 cells cultured using four different culture conditions. Data are represented as the mean of three biological replicates of three reporter models.

Severe DNA damage may decrease cellular viability, and although this is a non-specific adverse outcome, it may be a sensitive general readout of toxicity. To evaluate cell death induction upon exposure to DNA damage inducing agents in the different HepG2 culture conditions, cell viability was determined by evaluating the PI-positive fraction and ATP content (Supplementary Figures 3 and 4, available at *Mutagenesis* Online) followed by hierarchical clustering of all the data (Figure 4). Clustering of both cell death end points showed in general that HepG2 cells exposed for a longer duration (4-day repeated in 3D or 72 h in 2D), clustered together based on the medium type that was used. Most cell death was seen with mitomycin C exposure, where HepG2 cells grown in 3D with normal DMEM medium were most sensitive with an IC_50_ of 1.58 µM. When cells were grown in 2D with normal DMEM medium, their tolerance of mitomycin C was about 4-fold higher, with an IC_50_ of 6.31 µM. HepG2 cells grown in 2D with AAGLY medium were less sensitive, showing only reduction in ATP content at the highest concentration with 72-h exposure (IC_50_ of 20.43 µM). Cell death was also observed upon cisplatin treatment at concentrations higher than 10 µM, but this was only the case for HepG2 cells grown in normal DMEM medium either in 2D or 3D. No cell death was seen with cisplatin when cells were grown in AAGLY medium. Brequinar only induced cell death at the highest concentration of 100 µM in HepG2 cells grown in 3D in both medium types or in 2D in combination with AAGLY medium. When cells were cultured in 2D using normal DMEM medium, no cell death was seen for brequinar, suggesting that an improved hepatic phenotype is needed to identify brequinar-mediated adversity. Aflatoxin B1 showed highest cell death induction in HepG2 cells grown in 3D with DMEM/F12 medium at concentration levels of 2.15 µM or higher. Cells grown in 2D with normal DMEM medium were less sensitive showing adversity at 10 µM or higher. When AAGLY medium was used no cell death could be observed during aflatoxin B1 exposure, although DDR activation was seen. Gemcitabine did not show cell death induction in all the models.

### Shift in sensitivity for chemical-induced DDR activation

**Fig. 5.**
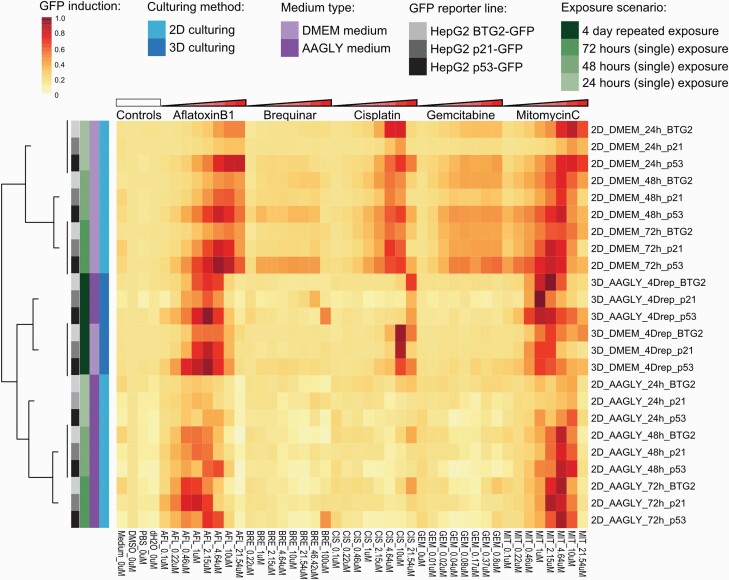
Hierarchical clustering of the reporter activation. Hierarchical clustering (Euclidean distance with Ward’s distance) based on fold change GFP reporter data, including seven compounds, at eight concentrations, in three different reporter models (p53, p21 and BTG2-GFP) at four different culture conditions. *N* = 3.

To assess genotoxic mechanisms that may underlie the observed compound-induced cytotoxicity, we systematically compared the difference in DNA damage signalling activation among the HepG2 models upon chemical exposure. We created a hierarchical clustering of the activation of the three DNA damage GFP reporters at all test conditions from which the shifts in sensitivity between the different conditions can be clearly observed (Figure 5). The dendrogram on the left indicated that reporter data of the HepG2 cultured in the normal DMEM medium in 2D separated from the other, more metabolically active, culture conditions. The reporter data of HepG2 cultures in 3D with the two different media types showed the highest resemblance with each other.

When examining individual compounds, aflatoxin B1 induced DNA damage reporters (BTG2, p21 and p53) in all culture conditions. However, the more metabolically active models, e.g. 2D HepG2 cells cultured in AAGLY medium, were more sensitive as the induction was already observed at the lowest concentration of 0.1 µM aflatoxin B1 (Supplementary Figure 2, available at *Mutagenesis* Online). Interestingly, for the HepG2 reporters cultured in 2D, the reporter induction by aflatoxin B1 was more prominent, up to ~4.5-fold, when measured at later time points (48 and 72 h). In general, the BTG2-GFP and p53-GFP reporters were most sensitive towards aflatoxin B1-induced DNA damage.

Brequinar, an inhibitor of pyrimidine synthesis, only led to mild induction of the DNA damage reporters, of which p53 was most sensitive (Supplementary Figure 2, available at *Mutagenesis* Online) in particular with the 2D set-up using normal DMEM medium resulting in maximal ~3-fold higher induction compared to other reporters. Interestingly, no DDR was observed at 24 h after brequinar exposure. In the 4-day repeated dosing scenario in 3D in combination with AAGLY medium, brequinar induced a DDR (p21 and p53 activation) at the highest concentration, but this was not seen when using normal DMEM medium.

All DNA damage reporters showed a very clear concentration-dependent activation upon exposure to the direct mutagen cisplatin in 2D with normal DMEM medium at 0.46 µM or higher. However, when AAGLY medium was used or when the cells were cultured in 3D, this effect was much less pronounced (see also Supplementary Figure 2, available at *Mutagenesis* Online) where clear activation was only seen at 10 µM or higher. It is likely that cell cycle progression is required for cisplatin-induced DNA damage effects. Similar results were obtained for gemcitabine (see also Supplementary Figure 2, available at *Mutagenesis* Online). Only in proliferative culture conditions (2D with normal DMEM medium), the genotoxic effects of gemcitabine were revealed, which seem to be almost absent in the other culture conditions.

The last compound tested, mitomycin C, had both a direct as well as an indirect DNA-damaging effect. HepG2 3D spheroids in combination with repeated dosing were most sensitive to identify DDRs by mitomycin C showing the highest reporter induction at lower concentrations compared to the other models.

**Fig. 6.**
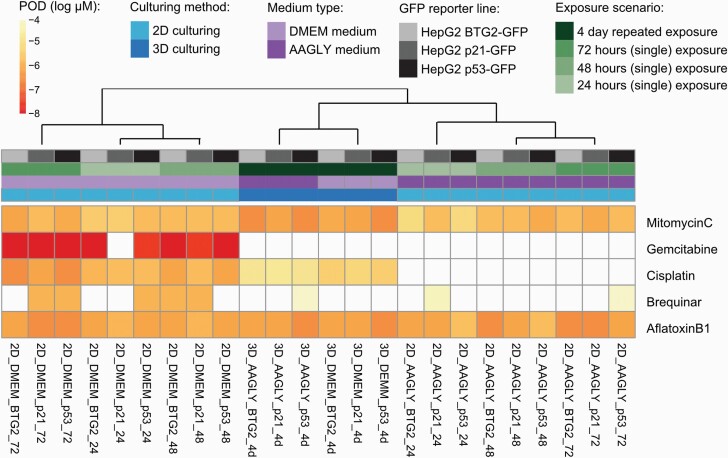
Hierarchical clustering of the reporter activation PODs. Hierarchical clustering (Euclidean distance with Ward’s distance) based on the PODs of GFP reporter intensity concentration–response curves.

To get more insight in the sensitivities of our DDR reporters in the different culture conditions, we calculated for each compound and condition the PoD values of reporter activation. PoD values were defined as the lowest compound concentrations resulting in significant induction of reporter activity. Hierarchical clustering of the PoD values for the three DDR stress pathway reporters (p21-, p53- or BTG2-GFP) confirmed their similarities in response, and showed that drug sensitivity was largely dependent on culture conditions (Figure 6). By culturing the HepG2 cells in 2D with normal DMEM medium, PODs could be determined for all compounds. This model seemed to be especially sensitive for gemcitabine, for which very low PoD values were calculated of 0.028 µM or lower. This compound did not induce a quantifiable genotoxic effect in the other models. 3D culturing conditions with repeated dosing increased the sensitivity of the reporters for the genotoxic effects of mitomycin C, as in these conditions the lowest PoD was 0.32 µM mitomycin C while for 2D conditions the lowest defined PoD was 0.74 µM. Genotoxic effects of aflatoxin B1, a compound that required bioconversion to a genotoxic metabolite, could be best picked up using the 3D culturing or by using AAGLY medium. The delayed DDR after brequinar treatment was best measured at the later time points in the 2D cultures, which could not be picked up with the 4-day repeated dosing scenario in the 3D set-up. DNA-damaging effects at low concentrations of cisplatin were detected in the 2D culture with normal DMEM medium giving PODs of 0.32–1.83 µM. In 3D, this effect was also observed but at higher concentrations ranging from 3.35 to 17.2 µM, especially in combination with AAGLY medium. By using the combination of 2D culturing and AAGLY medium, cisplatin-induced DDR could not be detected.

When comparing PODs of the DNA damage reporter activation (Figure 6; Supplementary Figure 2, available at *Mutagenesis* Online) with the compound concentrations at which the cell viability was affected (Figure 4; Supplementary Figures 3 and 4, available at *Mutagenesis* Online), we found that the readout for DDR reporters is much more sensitive. Typically, the DNA damage BAC-GFP reporter cell lines were activated at 10–50 times lower concentrations as compared to the concentrations at which cell death (PI) and cell viability (ATP-lite) were observed.

## Discussion

In this study, we systematically compared the impact of differentiation-inducing 3D culturing and AAGLY medium on the detection of (in)direct genotoxicity in HepG2 liver cancer cells. For this purpose we used three fluorescent HepG2 reporters for p53 pathway activation in the DDR: p53-GFP, BTG2-GFP and p21-GFP. The key findings of our study are: (i) HepG2 DDR reporters are more sensitive to DNA-damaging agents than HepG2 cell death or viability assay; (ii) proliferating HepG2 DDR reporters cultured under standard 2D conditions are generally sensitive to diverse DNA-damaging agents with diverse modes of action; (iii) differentiated HepG2 DDR reporters cultured in 3D or AAGLY medium are not sensitive to the genotoxic drugs cisplatin, brequinar and gemcitabine; (iv) 2D and 3D HepG2 DDR reporters with non-proliferative differentiated phenotypes are more sensitive towards genotoxicants that require bioactivation by P450 enzyme systems. These findings suggest that a genotoxicity testing strategy with our panel of HepG2 DDR reporters should consist of (i) proliferating HepG2 DDR reporters in normal culture medium followed by (ii) differentiated HepG2 DDR reporters using AAGLY medium with enhanced expression of P450 mimicking primary hepatocyte levels.

The DDR reporter activities induced by cisplatin, gemcitabine and brequinar were mainly observed in normal proliferating 2D cells. Since the genotoxic effects of these drugs depend on replication, this is likely explained by the fact that HepG2 cells cease to proliferate in 3D or AAGLY medium (17,18). Many other known genotoxic compounds have similar replication-dependent modes of action, which advocates for the use of 2D HepG2 reporter systems in the early phase of genotoxic hazard characterisation.

HepG2 DDR reporters cultured under conditions with enhanced differentiation characteristic were most sensitive towards aflatoxin B1. This suggests that differentiation of HepG2 DDR reporters would in general increase the sensitivity to compounds that require metabolism to become genotoxic. The genotoxic effect of aflatoxin B1 depends completely on liver-specific metabolisation to its reactive forms aflatoxin B1-8,9-exo-epoxide, 8,9-dihydroxy-8-(N7) guanyl-9-hydroxy aflatoxin B1 and aflatoxin B1 formaminopyrimidine (38). Interestingly, for the HepG2 lines cultured in 2D the reporter induction by aflatoxin B1 was more prominent when measured at later time points (48 and 72 h), which might suggest that the reactive metabolite of aflatoxin B1 (AFB1-8,9-epoxide) is accumulating over time. *CYP3A4* and *CYP3A7* were highly expressed in differentiated HepG2 cell culture conditions in the presence of AAGLY medium. Indeed, our p53 pathway reporters were most sensitive to aflatoxin B1 in AAGLY medium. The advantage of these differentiated HepG2 test systems is that it allows repeated dosing regimens mimicking better real-life exposures. This is generally hampered in proliferating 2D-cultured HepG2 cells. Besides aflatoxin B1, also mitomycin C was able to induce strong DDR reporter activity in differentiated HepG2 test systems. Which makes sense as mitomycin C can directly damage DNA through DNA cross-linking and inhibit both DNA replication as well as transcription. It can also indirectly induce DNA damage via cytochrome P450-mediated redox cycling and reactive oxygen species (ROS) formation (32). What is the overall added value of physiologically more relevant liver model systems for the assessment of genotoxicity? To answer this question, there are a couple issues to consider. To begin with, our p53 pathway reporters do not directly measure DNA damage itself, but rather the cellular response to it. This allows sensitive and early detection of low levels of DNA damage, which justifies benchmarking this reporter system against other genotoxicity test systems using PoD analyses. A recent evaluation of cisplatin genotoxicity using the high-throughput CometChip assay shows a PoD of 6.3 µM in PHH (39), which is considerably higher than the PoD values of 0.32–1.83 µM obtained with our DDR reporters. Thus, HepG2 BAC-GFP DDR reporter cell lines may be more sensitive than established genotoxicity assays, which should be further evaluated using a broader spectrum of genotoxicants. Moreover, the GFP-protein fusions also make it possible to follow dynamics of p53 pathway activation, which is an important determinant of cell fate (32). For the HepG2 DDR reporters grown in 2D we have evaluated reporter activation at three time points, within a 3-day period, but could be extended using increased time resolution to more accurately map the DDR activation dynamics upon exposure. Together, our reporters give a robust representation of p53 pathway activation, because protein expression levels of p53 and its target genes *BTG2* and *CDKN1A* are similarly induced by DNA damage and highly co-regulated (40). However, the p53 pathway may also be upregulated by non-genotoxic compounds (41). Dihydroorotate dehydrogenase inhibitors such as brequinar, for which we only detected reporter activity in standard 2D culture, have recently been shown to increase p53 protein expression without preceding or concomitant induction of more direct markers of DNA damage such as ataxia-telangiectasia mutated (ATM) or ATM and Rad3-related (ATR) phosphorylation (42). Mechanistically, the increased p53 protein expression was suggested to be a consequence of accumulation of cancer cells in S-phase. Thus, it is possible that some of the activity of the standard-2D culture does not reflect actual DNA damage. Since HepG2 reporter lines cultured in a more differentiated state will undergo G0/G1 arrest, short-term effects on their cell cycle distribution seem less likely. Whether there may be other non-genotoxic triggers for p53 reporter activity in differentiated HepG2 cells remains to be determined.

Some compounds cause the cellular formation of ROS, which can have an effect on the stability of the DNA and therefore have a delayed genotoxic effect (43). Furthermore, there seems to be cross talk from the inflammation to the DNA damage pathway and vice versa (44). By extending the reporter panel with the previously generated Nrf2 (oxidative stress) and NF-κB (inflammation) pathway reporter cell lines (14) it may be possible to discriminate between direct and indirect genotoxic modes of action (13).

In addition, we tested only a few compounds, and we do anticipate that various compounds at risk for genotoxicity may require bioactivation through cytochrome P450 enzyme system, which are insufficiently expressed in HepG2 cells in standard 2D culture. For an accurate estimation of the performance (sensitivity, predictivity and accuracy scoring) of the 2D and 3D systems a larger screen, including different classes of genotoxic and non-genotoxic compounds should be performed (45). More importantly, the added value of cell culture-based assays for genotoxicity over classical assays such as the Ames test in bacteria lies not only in the detection of indirect genotoxicity alone. The ultimate goal is to use cell culture-based toxicity assays as quantitative, predictive tools for adverse outcome (1). For that purpose, it is essential to use model systems that accurately reflect the human bioactivation *in vivo* response to toxic chemicals.

Taken together, our data indicate that standard 2D HepG2 models are capable of identifying diverse mechanisms of DNA responses but are not optimally equipped to effectively detect indirect genotoxicants that require bioactivation through drug-metabolising enzymes. The reverse is the case for differentiated HepG2 cell culture systems that are highly insensitive for genotoxicants that impact on DNA replication, but seem particularly more sensitive for genotoxicants that require bioactivation. Therefore, we propose a tiered testing strategy with the combination of both standard 2D HepG2 cells and differentiated HepG2 cells cultured in 2D or 3D in AAGLY medium. A broader follow-up screen based on a larger and unbiased set of compounds is necessary to assess the true potential of such a combined screening strategy.

## Supplementary Material

geab031_suppl_Supplementary_Figure_S1

geab031_suppl_Supplementary_Figure_S2

geab031_suppl_Supplementary_Figure_S3

geab031_suppl_Supplementary_Figure_S4

geab031_suppl_Supplementary_Table_S1

## Data Availability

The data underlying this article will be shared on reasonable request to the corresponding author.
