# Supplementary figures and images for: Towards an advanced testing strategy for genotoxicity using image-based 2D and 3D HepG2 DNA damage response fluorescent protein reporters
Source: Mutagenesis. 2021 Aug 27;37(2):130–42. doi: 10.1093/mutage/geab031 (PMC9071099; doi:10.1093/mutage/geab031)

# Dose response DDR reporter genes – Cisplatin

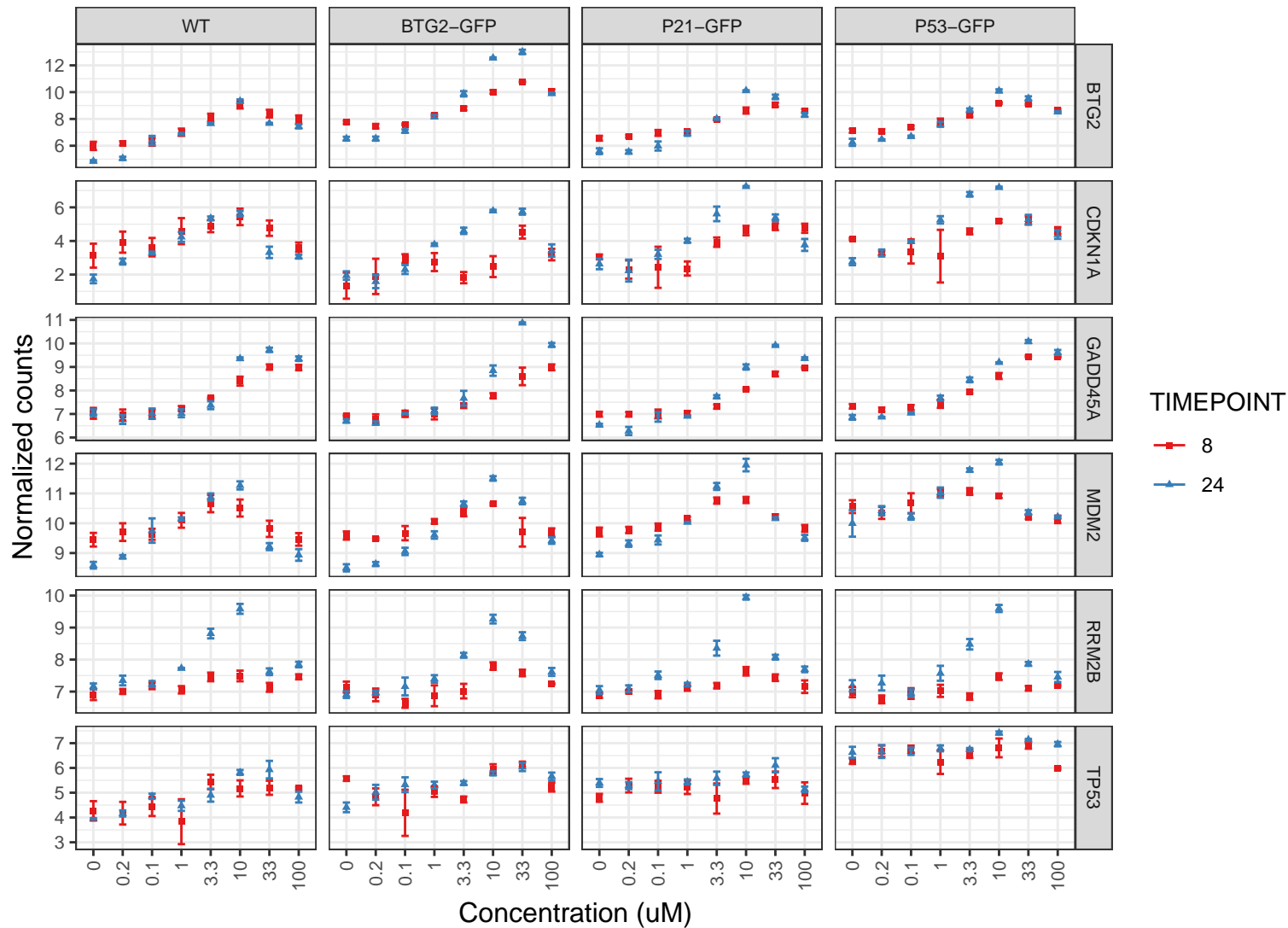

Supplement: geab031_suppl_Supplementary_Figure_S1 [file geab031_suppl_supplementary_figure_s1.pdf]

# HepG2 BTG2-GFP

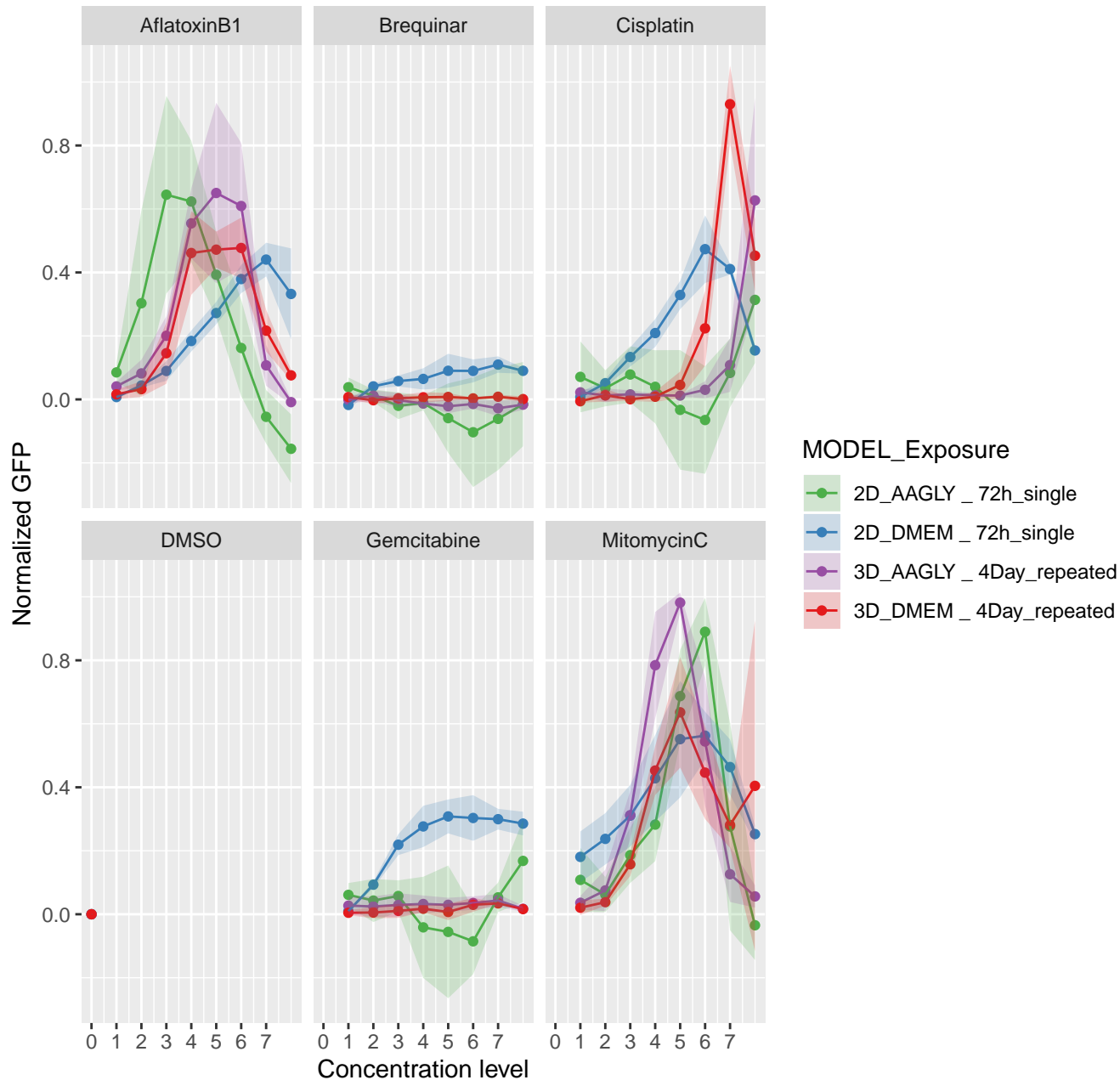

# HepG2 p21-GFP

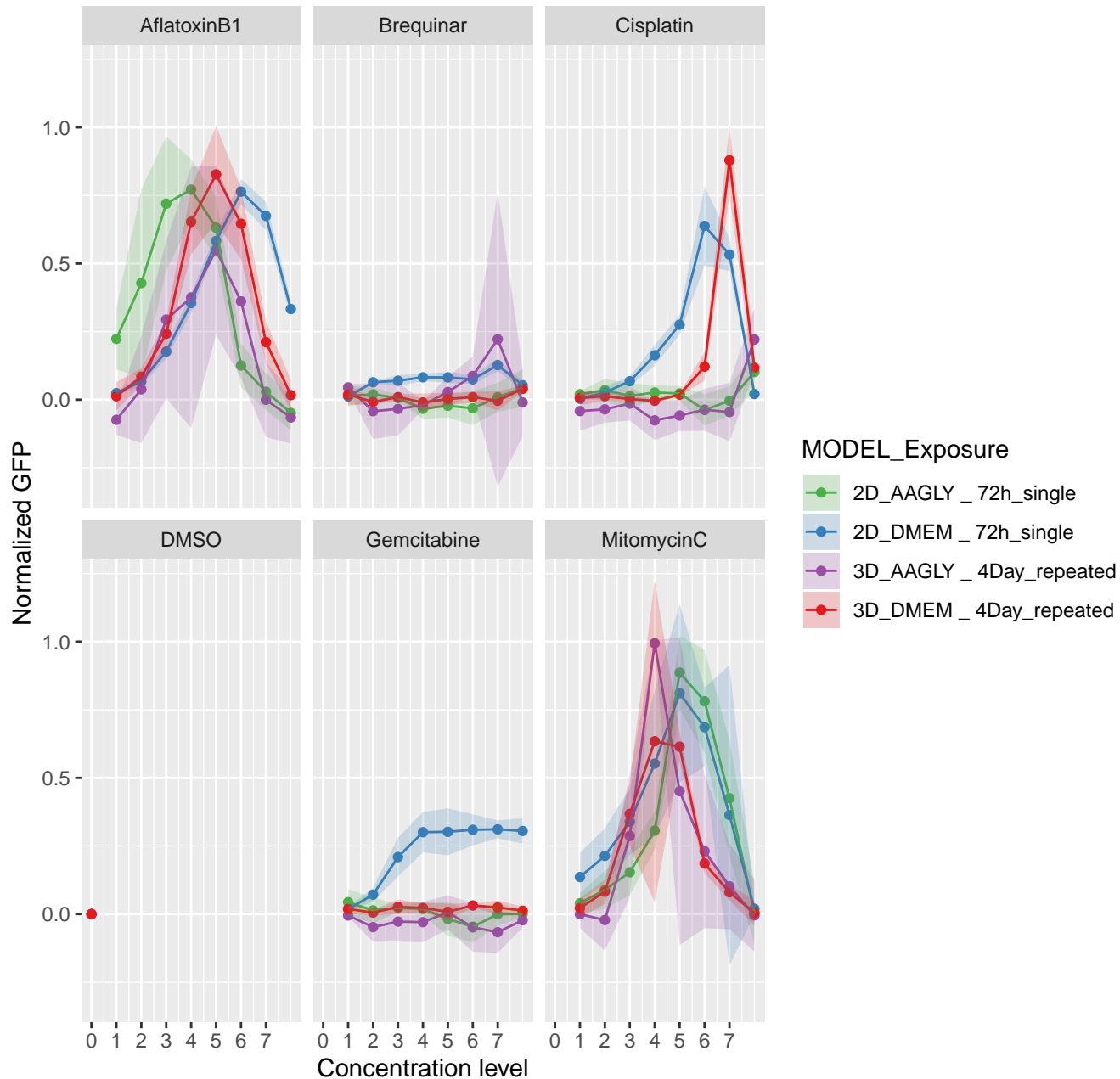

# HepG2 p53-GFP

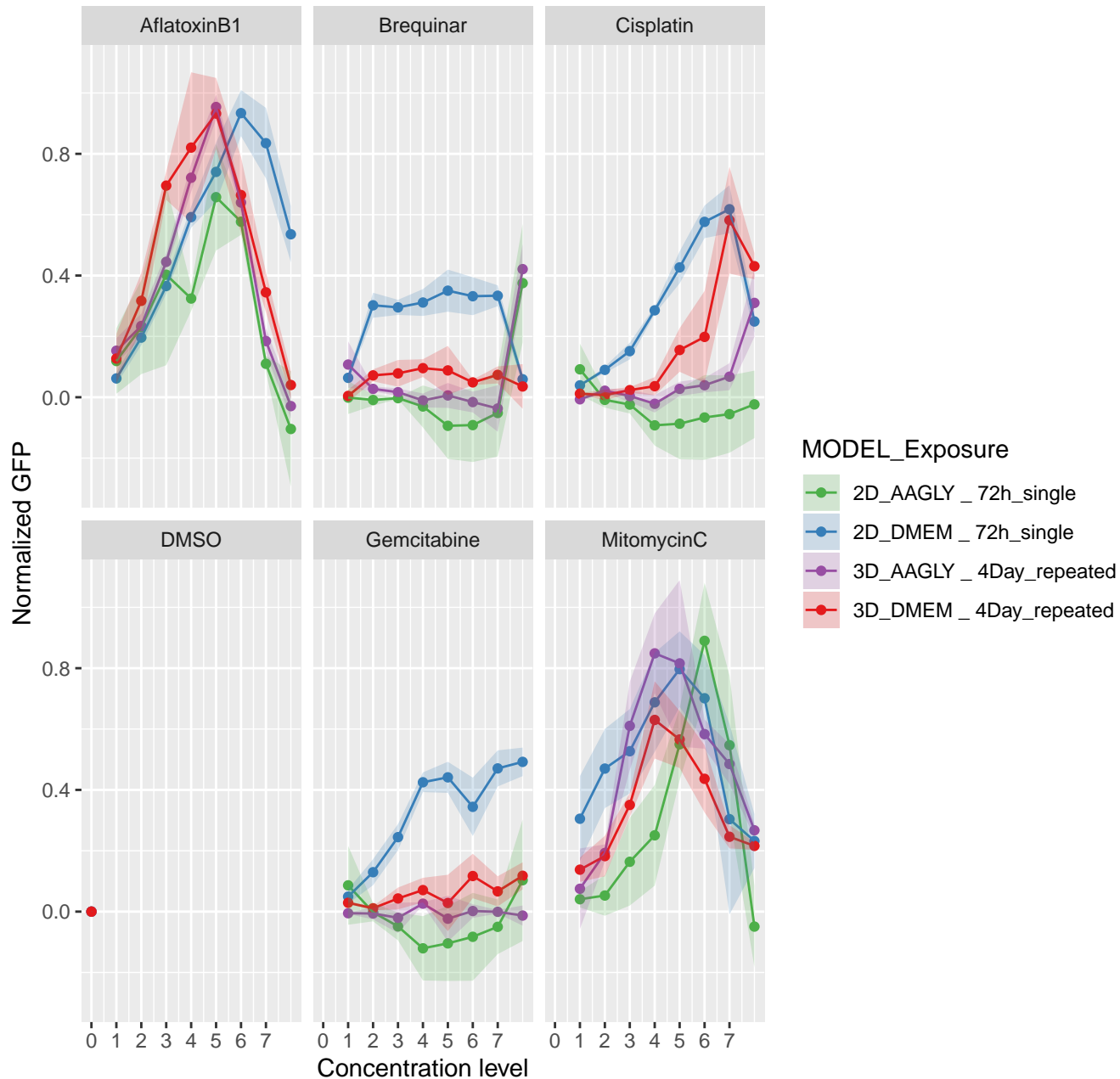

Supplement: geab031_suppl_Supplementary_Figure_S2 [file geab031_suppl_supplementary_figure_s2.pdf]

# Fraction PI over all reporters

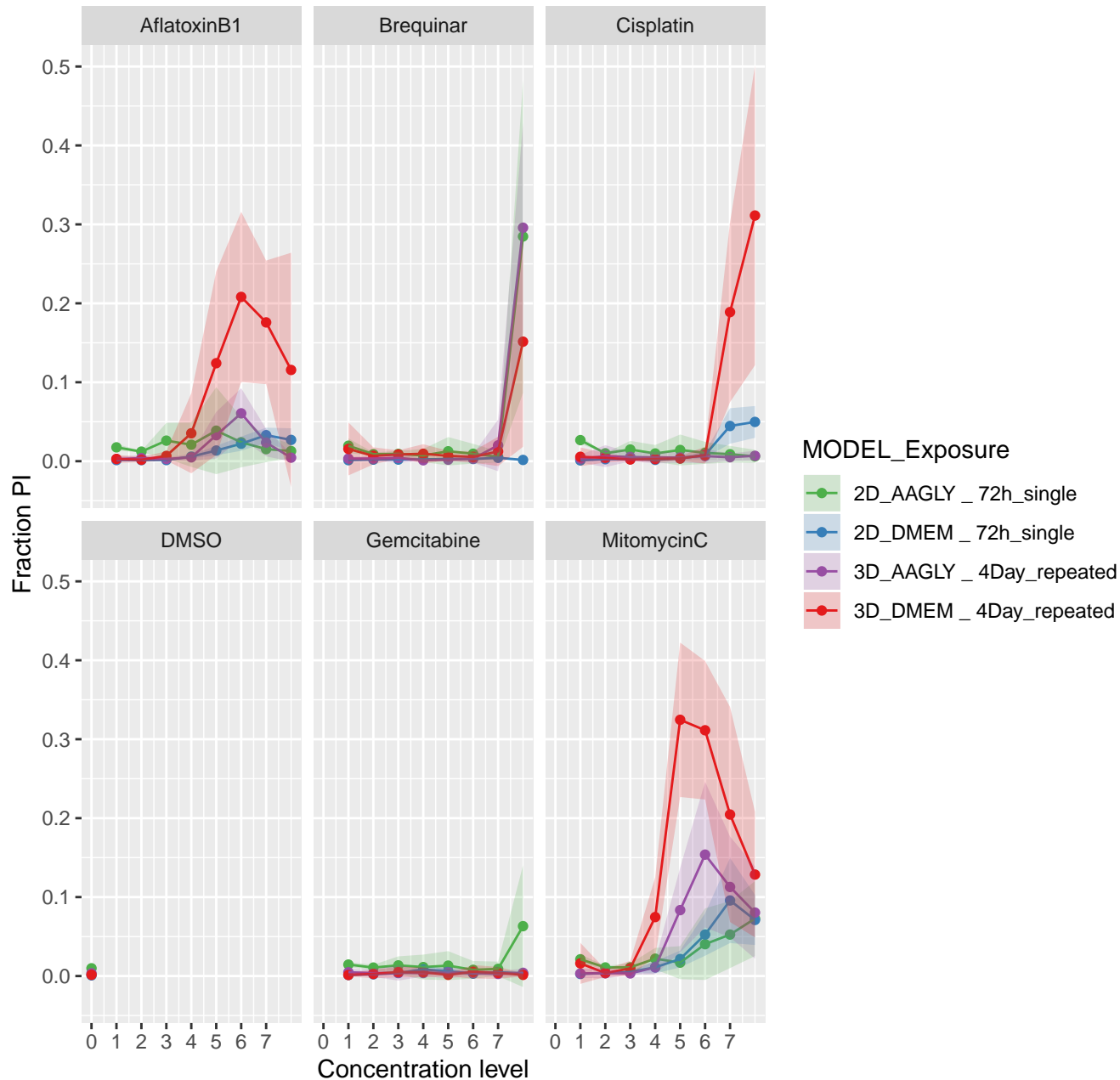

Supplement: geab031_suppl_Supplementary_Figure_S3 [file geab031_suppl_supplementary_figure_s3.pdf]

# Relative ATP content over all reporters

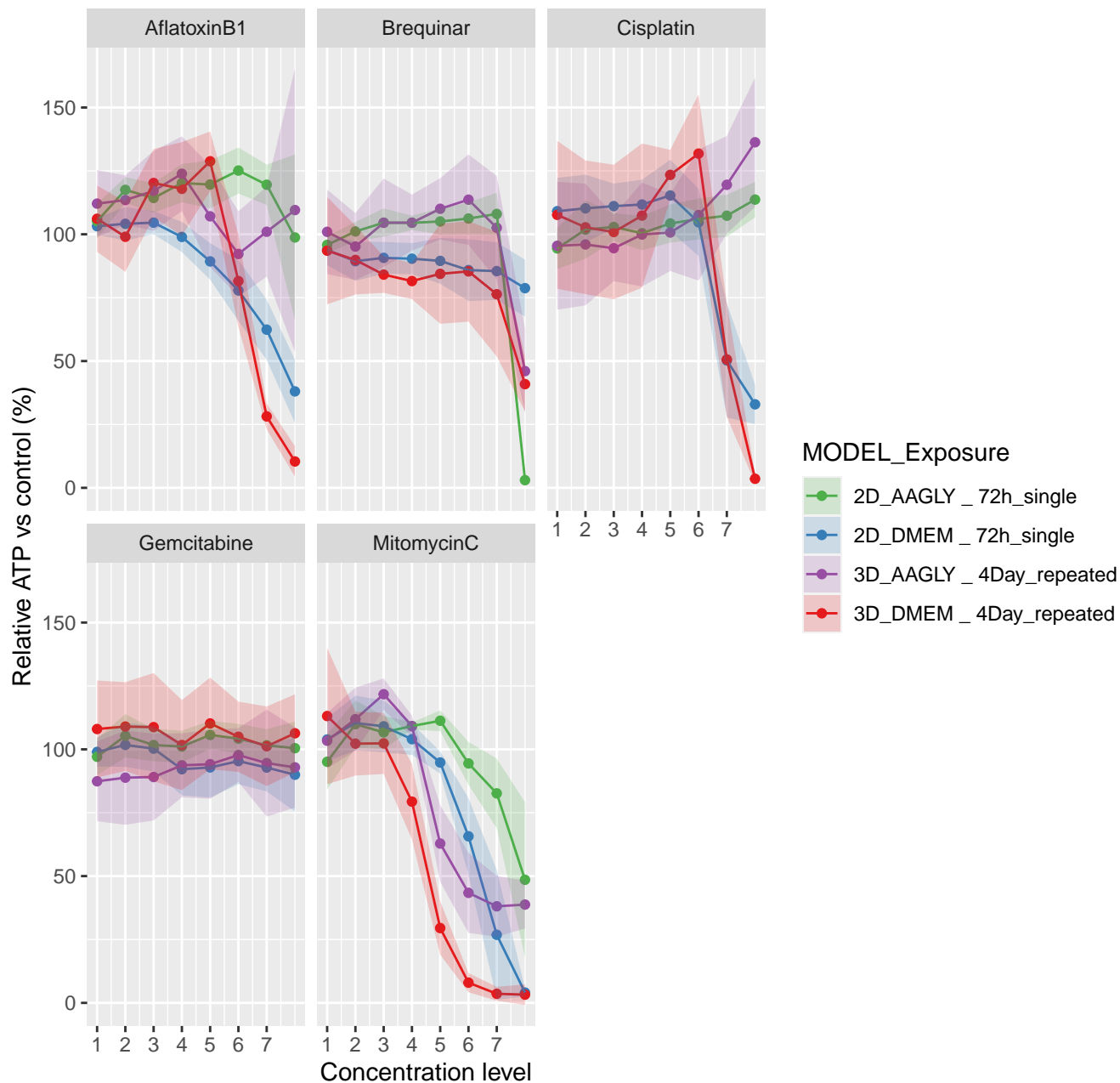

Supplement: geab031_suppl_Supplementary_Figure_S4 [file geab031_suppl_supplementary_figure_s4.pdf]
