# Supplementary material for: Towards an advanced testing strategy for genotoxicity using image-based 2D and 3D HepG2 DNA damage response fluorescent protein reporters
Source: Mutagenesis. 2021 Aug 27;37(2):130–42. doi: 10.1093/mutage/geab031 (PMC9071099; doi:10.1093/mutage/geab031)
Supplement: geab031_suppl_Supplementary_Table_S1 [file geab031_suppl_supplementary_table_s1.pdf]

#	Oligo Name	Gene	Marker	Sequence 5' to 3' (include modification codes if applicable)
1	H_AAT1_fw			ACTGGGGTGACCTTGGTTAAT
2	H_AAT1_rv	AAT1	Hepatocytes	GACGGCATTGTCGATTCACTG
3	H_AFP_fw			TGAGCACTGTTGCAGAGGAG
4	H_AFP_rv	AFP	Fetal hepatocytes	GTGGTCAGTTTGCAGCATTC
5	H_ALB_fw			ATGCTGAGGCAAAGGATGTC
6	H_ALB_rv	Albumin	Hepatocytes	AGCAGCAGCACGACAGAGTA
11	H_CYP1A2_fw			CTTTGACAAGAACAGTGTCCG
12	H_CYP1A2_rv	CYP1A2	Hepatocytes	AGTGTCCAGCTCCTTCTGGAT
13	H_CYP1B1_fw			ACCAGGTATCCTGATGTGCAGAC
14	H_CYP1B1_rv	CYP1B1	Hepatocytes	AGGTGTTGGCAGTGGTGGCATGAG
17	H_CYP3A4_fw			tTCCTCCCTGAAAGATTCAGC
18	H_CYP3A4_rv	CYP3A4	Hepatocytes	GTTGAAGAAGTCCTCCTAAGCT
21	H_CYP3A7_fw			AGATTTAATCCATTAGATCCATTCG
22	H_CYP3A7_rv	CYP3A7	Hepatocytes	AGGCGACCTTCTTTTATCTG
27	H_GAPDH_fw			TCAAGAAGGTGGTGAAGCAGG
28	H_GAPDH_rv	GAPDH	Housekeeping	ACCAGGAAATGAGCTTGACAAA
31	H_HNF4a_fw			ACTACGGTGCCTCGAGCTGT
32	H_HNF4a_rv	HNF4α	Hepatocytes	GGCACTGGTTCCTCTTGTCT
35	H_NTCP_fw			ATCGTCCTCAAATCCAAACG
36	H_NTCP_rv	NTCP	Hepatocytes	CCACATTGATGGCAGAGAGA
41	H_RLP19_fw			ATTGGTCTCATTGGGGTCTAAC
42	H_RLP19_rv	RLP19	Housekeeping	AGTATGCTCAGGCTTCAGAAGA
49	H_CYP2A6_Fw			TTTTGGTGGCCTTGCTGGT
50	H_CYP2A6_Rv	CYP2A6	Hepatocytes	GGAGTTGTACATCTGCTCTGTGTTCA
51	H_CYP2D6_Fw			CCTACGCTTCCAAAAGGCTTT
52	H_CYP2D6_Rv	CYP2D6	Hepatocytes	AGAGAACAGGTCAGCCACCACT
55	H_CYP2E1_Fw			AATGGACCTACCTGGAAGGAC
56	H_CYP2E1_Rv	CYP2E1	Hepatocytes	CCTCTGGATCCGGCTCTCATT
47	H_UGT1A1_fw			CAGCAGAGGGGACATGAAAT
48	H_UGT1A1_rv	UGT1A1	Hepatocytes	ACGCTGCAGGAAAGAATCAT
59	H_CYP2C19_Fw			CAACAACCCTCGGGACTTTA
60	H_CYP2C19_Rv	CYP2C19	Hepatocytes	GTCTCTGTCCCAGCTCCAAG
